# The Kentucky SimSmoke Tobacco Control Policy Model of Smokeless Tobacco and Cigarette Use

**DOI:** 10.34172/ijhpm.2020.187

**Published:** 2020-10-27

**Authors:** Luz María Sánchez-Romero, Zhe Yuan, Yameng Li, David T. Levy

**Affiliations:** Lombardi Comprehensive Cancer Center, Georgetown University, Washington, DC, USA.

**Keywords:** Smokeless Tobacco, Cigarette, Tobacco Policy, Simulation Model, Kentucky

## Abstract

**Background:** Smokeless tobacco (SLT) prevalence was decreasing in Kentucky before 2007, but has since increased. This study examines the impact of policies on cigarette and SLT use by applying the *SimSmoke* tobacco control policy simulation model.

**Methods: **Using data from the large-scale Tobacco Use Supplement of the Current Population Survey (TUS-CPS) and information on state-specific tobacco policies, *Kentucky SimSmoke* is updated and extended to incorporate exclusive SLT and dual cigarette and SLT use. The model is validated using survey data through 2017. The model was used to estimate the impact on smoking and SLT prevalence and attributable deaths of policies implemented between 1993 and 2018 and the impact of stronger future policies implemented in 2018 and maintained through 2060.

**Results:** *SimSmoke* generally reflects trends in exclusive cigarette use from the TUS-CPS and the Behavioral Risk Factor Surveillance System (BRFSS), but underestimated the increase in SLT prevalence in recent years. *SimSmoke* projects that policies implemented between 1993 and 2018 reduced male and female cigarette use by 23.7% and 23.0%, and male and female SLT use by 4.9% by 2018, averting 9018 tobacco-attributable deaths by 2018, increasing to 89 547 by 2060. The largest reductions in cigarette and SLT use were attributed to cigarette price increases. Strengthening tobacco control policies could reduce smoking prevalence by 41% and 40%, and reduce SLT prevalence by 33% and 25% for males and females by 2060.

**Conclusion: **Our results suggest that cigarette-oriented policies were effective in reducing SLT use but have been less successful in recent years. Future use rates can be further reduced through more restrictive statewide policies, which also target non-combustible nicotine products.

## Background

**Key Messages**

**Implications for policy makers**

- Kentucky has relatively weak policies, and stronger tobacco control policies are needed.
- In particular, policies in Kentucky need to be directed at cigarette use, including higher cigarette taxes, comprehensive smoke-free air laws, and media campaigns
- Marketing restrictions, media campaigns and youth access restrictions should also be directed at smokeless tobacco (SLT) use.
- The tobacco industry should be monitored.

**Implications for the public**

While the landscape for nicotine delivery products has dramatically changed in the last ten years, some lessons can be gleaned from our results. First, with cigarettes as the dominant form of nicotine delivery, cigarette-oriented policies are an effective means of reducing the use of nicotine delivery products. Well targeted policies and regulations, such as cigarette and smokeless tobacco (SLT) tax increases and media campaigns, will be needed to achieve the national Healthy People 2020 objective of less than 1% SLT use. However, with SLT use increasing in recent years, policies directed at SLT may also play a role. With cigarette manufacturers having acquired major SLT firms, it is important to monitor the role of the cigarette industry, as it has strong incentives to protect the high profits from cigarettes. Their incentives are to encourage dual use rather than having individuals switch to SLTs or quit all tobacco use. Similar implications may be expected for e-cigarettes.

Kentucky has one of the highest rates of cigarette smoking prevalence in the United States at about 24.6% in 2017,^1^ compared to 14.0% for the United States as a whole.^2^ Kentucky also has some of the weakest state-level tobacco control policies,^3,4^ with tax rates among the lowest of all states, limited smoke-free legislation in some cities, and tobacco control spending (less than 10% of the CDC [Centers for Disease Control and Prevention] recommended minimum) ranked among the lowest 25% of states in 2018.^5^ In addition to high smoking prevalence, Kentucky also has a high rate of smokeless tobacco (SLT) prevalence. The Behavioral Risk Factor Surveillance System (BRFSS) found that Kentucky SLT prevalence was 7.6% (almost 14% among males)^1^ in 2017 compared to the 4.1% national average.^6-8^ Also, any past-30 day use of SLT products among Kentucky high schoolers was estimated at 10.6% in 2017, with the national average rate at 5.5%.^9^

 While much of SLT use takes the traditional forms of snuff and chewing tobacco, new forms of smokeless, such as different varieties of snus and other oral tobacco products, have also become available in recent years.^10,11^ With lower health risks than cigarettes,^12-14^ these products may reduce the harms associated with cigarettes. Their impact will depend on the relationship of SLT use to smoking.^15^ If SLT use reduces smoking initiation or helps smokers who would not have otherwise quit to either quit or entirely switch to SLTs, then public health improves. On the other hand, SLT use may encourage youths to take up tobacco use or discourage smokers who would have otherwise quit from actually quitting, and thus have a harmful public health impact. In particular, smokeless can act as a complementary product to smoking and help maintain smoking through dual use. While we focus on SLT use, similar concerns arise with e-cigarette use.^15^ With a shorter history than SLT use, less is known about e-cigarette use. A better understanding of patterns of SLT use, especially among youth and young adults, and of the effects of tobacco control policies on SLT use may be helpful in understanding the public health impact of e-cigarette vis-à-vis cigarette use and the potential impact of policies on that use.

*The Kentucky SimSmoke* tobacco control simulation model is used to examine the impact of past and stronger future tobacco control policies on Kentucky’s tobacco users. This paper updates a previous version of *Kentucky SimSmoke* (1993-2006)^3^ to consider smoking prevalence through 2018 and incorporate exclusive and dual SLT use. The new model distinguishes exclusive and dual SLT use and considers the impact of implementing individual and combined policies on smoking and SLT prevalence and tobacco-attributable deaths in Kentucky.

## Methods

 The *Kentucky SimSmoke, *a discrete first-order Markov model, starts with the 1993 population by age and gender. The population is distributed into seven smoking categories: never tobacco users, current and former exclusive smokers, current and former exclusive SLT users, and current and former dual users. Over time, cigarette and SLT users at each age change through four principal modules: population, tobacco use, tobacco-attributable deaths, and tobacco regulation policies. The model is based on a previously developed model for the United States.^16^ Further discussion of the new Kentucky model is found in a longer supplementary report.^17^

###  Population

 The population evolves through births, deaths and migration. Kentucky population data was obtained from the Census Bureau for 1993 to 2015,^18^ with projections through 2040.^19^*SimSmoke* uses the actual population for ages 0-14, with the population for 2040-2060 linearly extrapolated from 2030-2040. At later ages, the population evolves via mortality and migration rates,^20^ and was adjusted to within 7% of 2015 Kentucky population estimates.

###  Tobacco Use

 Individuals evolve from never tobacco users to current exclusive smokers, exclusive SLT, or dual users through cigarette, SLT, and dual initiation. Before age 14, the population is considered to consist entirely of never smokers. From age 15, a percentage of each age group transitions to current tobacco users through smoking and SLT initiation rates. Current exclusive smokers, exclusive SLT users, or dual users may quit and thereby become former users. Former exclusive smokers through cessation may return to smoking through relapse and similarly for former exclusive SLT users and former dual users. Former dual users do not relapse to exclusive tobacco users. A discrete time, first-order Markov process is employed to project smoking, SLT use, and dual use rates through initiation, cessation, and relapse.

 Baseline estimates of exclusive smoking, exclusive SLT, and dual use status by age and gender were obtained from the nationally-representative 1992/1993 Tobacco Use Supplement of the Current Population Survey (TUS-CPS).^21^ Current smoker is defined as having smoked more than 100 cigarettes in his/her lifetime and currently smoking either daily or some days. A question on SLT “use on a regular basis” is used to distinguish SLT users. Dual use is defined as those meeting the respective definitions of current smokers and SLT users. Due to the small female dual use prevalence (<0.1%), we do not consider this group. Former use is defined as those meeting their respective definitions of tobacco-use, but reporting no current use, and they are stratified into quit-years groups (<1, 1-2, 3-4, 5-9, 10-15, 16+ years). Since former smokers were not distinguished by former exclusive users and former dual users, they were estimated by multiplying former smoking prevalence by years quit by the ratio of current exclusive smokers or current dual users to total current smokers.

 The TUS-CPS does not provide sufficient information to distinguish initiation, cessation, and switching rates between SLT use and cigarettes, and the evidence on initiation and early transitions to SLT use is mixed.^22-26^ To measure initiation while incorporating cessation and switching, we calculate net initiation as the difference between 1993 prevalence at the current age and previous age. Since studies indicate limited switching between SLT use and cigarettes except at younger ages,^27-29^ we allow switching only via net initiation. Based on cigarette and SLT use peak rates, we allowed cigarette and SLT net initiation through age 27 for males and 34 for females.

 Tobacco cessation is explicitly modeled after the last age of net initiation. Smoker quit rates were obtained from the 1992/1993 TUS-CPS, measured as those who quit in the last year, but not the last three months.^30^ Because data were not available to estimate quit rates for exclusive SLT and dual users, we assumed that dual and exclusive SLT users quit and relapse rates by years quit are the same as for smokers^31-34^ based on findings that quit rates are at least as high among SLT and dual users as for cigarette users.^27-29,35,36^ Age, gender, and quit year-specific relapse rates are obtained from the US *SimSmoke.*^31-34^

###  Tobacco-Attributable Deaths

 Mortality rates were developed for each age, gender, and tobacco use category using the relative risks and prevalence rates of the categories and overall mortality by age and gender. Data on mortality relative risk for current and former smokers by age and gender were from the Cancer Prevention Study II.^33,37,38^ We assigned the same risks to exclusive cigarette and dual users, with risks declining for both former user categories with years quit.^33,37,38^ SLT users were assigned an overall mortality relative risk of 1.15 based on results from a large-scale US study.^13^

 Tobacco-attributable deaths were calculated as the number of users in each current and former smoking category multiplied by their excess mortality risk (ie, mortality rate of each current and former tobacco use group minus never smoker mortality rate) and then summed.

###  Policies

*SimSmoke* begins with policies at their 1993 levels and then incorporates the effect of policy changes between 1993 and 2018. *SimSmoke* includes tobacco prices (taxes), smoke-free air laws, mass media campaigns, marketing restrictions, health warnings, cessation treatment policies, and youth access. Table 1 shows the policy descriptions and effect sizes. Most policies are modeled as having direct reductions in prevalence rates (immediate impact) with the effect growing over time from changes in the initiation and cessation rates. Each policy effect size is estimated in terms of the percentage change relative to initial rates. All policy effect sizes are multiplicatively applied. Policy levels are based on the specific characteristics of each policy (eg, size of the tax, expenditures on media campaigns, coverage of smoke-free-air laws). Policy levels defined in Table 1, are based on those in effect in January of the corresponding year.

**Table 1:** Policy Inputs for Cigarette and Smokeless Tobacco *SimSmoke*

**Policy**	**Description**	Cigarette Effect Size*	**SLT Effect Size**
Tax policy			
Cigarette and SLT price	The effect of taxes is directly incorporated through average US price (including generics), with separate prices for cigarettes and SLT. The price elasticity (by age) is used to convert the price into effect sizes. The dual price computed as 4/5 of the cigarette price +1/5 SLT price.	Elasticities -0.4 ages 10-17 -0.3 ages 18-24 -0.2 ages 25-34 -0.1 ages 35-64 -0.2 ages 65 and above	Elasticities (exclusive SLT only) -0.4 ages 10-17 -0.3 ages 18-24 -0.2 ages 25 and above
Smoke-free air laws			
Worksite smoking ban, well-enforced	Ban in all indoor worksites in all areas, with strong public acceptance and enforcement of laws (reduced by 1/3 if allowed in ventilated areas and by 2/3 if allowed in common areas.	-6%	25% smoking effect size for exclusive SLT users and dual users
Restaurant smoking ban	Ban in all indoor restaurants in all areas (reduced by half if partial).	-2%	
Bars smoking ban	Ban in all indoor bars in all areas (reduced by half if partial).	-1%	
Other places bans	Ban in 3 of 4 of government buildings, retail stores, public transportation and elevators.	-1%	
Compliance	The government enforces and publicizes laws.	Effects reduced by 50% if no compliance	Same
Media campaigns			
Highly publicized media campaign	Campaign publicized heavily on TV and at least some other media, with a social marketing approach.	-6.50%	50% smoking effect size for exclusive SLT users; 100% smoking effect size for dual users
Moderately publicized media campaign	Campaign publicized sporadically on TV and at least some other media.	-3.25%	
Low publicity media campaign	Campaign publicized only sporadically in newspapers, billboards or some other media.	-1.63%	
Marketing restrictions			
Comprehensive marketing ban	Ban is applied to television, radio, print, billboard, in-store displays, sponsorships and free samples (all indirect marketing).	-5% prevalence, -8% initiation, +4% cessation	Same (100%) smoking effect size for exclusive SLT users and dual users
Total advertising ban	Ban is applied to all media television, radio, print, billboard, plus one indirect marketing.	-3% prevalence, -4% initiation, +2% cessation	
Weak advertising ban	Ban is applied to some of television, radio, print, billboard.	-1% in prevalence and initiation only	
Compliance	Based on exposure to advertisements.	Effects reduced by 50% if 0 exposure	
Health warnings			
Strong	Labels are large, bold and graphic and at least 30% of the package.	-4% prevalence, -6% initiation, +10% cessation	Same (100%) smoking effect size for exclusive SLT users and dual users
Moderate	Laws cover 1/3 of the package, not bold or graphic.	-2% in prevalence & initiation, +4 cessation	
Weak	Laws cover less than 1/3 of the package, not bold or graphic.	1% prevalence & initiation, +2% cessation	
Cessation treatment policies			
Availability of pharmacotherapies	Legality of NRT, Wellbutrin and varenicline.	-1% prevalence, +4% cessation	50% smoking effect size for exclusive SLT users; 75% smoking effect size for dual users
Proactive quitline	A proactive quitline with publicity through the media campaign with no cost NRT.	-1% prevalence, +6% cessation	
Treatment coverage	Payments to cover PT and behavioral cessation treatment.	-2.25% prevalence, +8% cessation	
Brief healthcare provider interventions	Asking about smoking, advising to quit and recommending effective cessation treatments.	-1% prevalence, +4% cessation	
All of the above	Complete availability and reimbursement of pharmaco- and behavioral treatments, quitlines, and brief interventions.	-5.74% prevalence, +29.44% cessation	
Youth access policies			
Strongly enforced & publicized	Compliance checks are conducted 4 times per year per outlet, penalties are potent and enforced, and with heavy publicity.	-16% initiation for ages 16-17 and -24% ages <16	50% smoking effect size for exclusive SLT users; 100% smoking effect size for dual users
Well-enforced	Compliance checks are conducted regularly, penalties are potent, and publicity and merchant training are included.	8% initiation for ages 16-17 and -12% ages <16	
Low enforcement	Compliance checks are conducted sporadically, penalties are weak.	2% initiation for ages 16-17 and -4% ages <16	
Vending machine ban	Total ban.	Enforcement effects increase by 8%	
Self-service ban	Total ban.	Enforcement effects increase by 4%	
Publicity	Community based and merchant publicity campaigns directed at youths.	Enforcement effects increase by 10%	

Abbreviations: SLT, smokeless tobacco; NRT, nicotine replacement therapy; PT, pharmacotherapy. * Unless otherwise indicated, the effects are on prevalence in the first year, and on initiation and cessation during the years that the policy is in effect. The effect sizes are based on articles referenced in the text.

 Cigarette and SLT price effects depend on price elasticities, prices through the tracking period, and tax changes in future years.^39^ Price elasticities are based on demand studies, which report similar elasticities for SLT and cigarettes.^40-42^ Cigarette prices are based on average Kentucky cigarette retail prices (including generics)^43^ from 1993 to 2018. SLT prices, the weighted average of the prices of chewing tobacco, snuff, and snus, are estimated by manufacturer prices, state and federal taxes, and wholesale and retail mark-ups.^44^ We adjusted post-2015 cigarettes and SLT prices by their respective state tax increases and inflation using the consumer price index.^45^ Price effects were weighted (75% cigarette, 25% SLT) for dual users.^17^ Federal and Kentucky cigarette excise taxes were increased in 2000, 2002, 2005, and 2009. Federal and Kentucky SLT taxes were increased in 2000, 2005, 2009, and 2010, then slightly decreased in 2013.^17^

 Smoke-free air lawsconsider the existence and enforcement of worksite, restaurant, pubs and bars, and other public place bans.^46^ The policy effect sizes (shown in Table 1) on exclusive SLT and dual use are assigned 25% of cigarette use.^42^ Before 2003, Kentucky had no significant state smoke-free airlaws, but since then, local restaurants, bars, and indoor workplace bans were implemented to cover about one-third of the state population.^47^ Based on estimates from the US model,^16^ we estimated 80% compliance for all users in 1993-2018.

*SimSmoke* considers tobacco control expenditures, which are usually for media campaigns.^48^ Based on evaluation studies of SLT-oriented media campaigns,^42^ the policy effect size for exclusive SLT use is 50% less than for exclusive and dual cigarette users. The levels are distinguished as high, medium, or low.^49^ Based on state expenditures,^50^ this policy level is set at low in 1993-1999, medium in 2000-2002, and low since 2003.

 Marketing restrictions are distinguished as high, medium, or low levels. In Kentucky, the policy was assigned a low level in all years with 80% exposure. The effect sizes for smokers and SLT users are the same.

 Health warnings depend on the size, location and whether they are graphic.^51^ Warnings on cigarette packages at a minimal level (<30% of the package) have not changed since first implemented in 1966. SLT package warnings were also at a minimum level until 2009. Since 2010, SLT packages have been required to display large text warnings covering at least 30% of two principal sides, raising the level to moderate.^42,52^

 Cessation treatment policies include the implementation and enforcement of four sub-policies: pharmacotherapy (PT) availability, financial coverage of treatments, quitlines, and brief interventions.^53^ Reviews of SLT cessation trials find mixed effects for pharmacotherapies, although slightly stronger effects for behavioral interventions.^42^ Nonetheless, SLT users utilize these resources at low rates.^54^ We set a 50% and 25% reduction of this policy effect for SLT and dual users, respectively, from the effect for smokers. Based on data from private and other healthcare program coverage in Kentucky, we estimate that 30% of the population is covered for PT and behavioral therapy from 1993 to 2009, increasing to 35% in 2010 with Medicaid coverage, and to 50% in 2014 with the Affordable Care Act. About 40% of smokers in all years were receiving brief interventions (asking about smoking, advising to quit and recommending effective cessation treatments) at appropriate levels from their healthcare providers.^55^ We also classify Kentucky as having no effective quitline until 2005, when the Kentucky Tobacco Quitline was implemented.^56^

 Strongly enforced and publicized youth access laws yield a larger reduction in youth smoking initiation for 10-15 year-olds than for 16-17 year-olds, and is enhanced by vending machines and self-service bans.^57^ Studies report lower compliance rates for SLT use than for cigarettes sales,^58,59^ and that tobacco youth access laws weakly affect youth SLT use.^60,61^We assign SLT users 50% and dual users 100% of the youth access policy cigarette effect sizes. Self-service and vending machine restrictions are set at 0% until 1997, 50% 1998-2008, and 100% since 2009. Enforcement was weak in 1997-1998 and increased to moderate since 1998.

###  Calibration and Validation 

 To calibrate *SimSmoke*, we compared its predictions of adult smoking and SLT prevalence by age and gender to corresponding estimates from the TUS-CPS data for 1996 and 1999. Based on these comparisons, we adjusted the first-year smoking cessation rates downward for ages 24-35 and upward for ages 55 and above.

 To validate the model, we compared *SimSmoke* predictions considering all policies implemented from 1993 to 2017 to rates of exclusive cigarette, exclusive SLT, and dual use from the 2002/2003, 2006/2007, 2010/2011 and 2014/2015 TUS-CPS survey. Results were also validated using gender-specific estimates from the BRFSS for overall smokers (exclusive cigarette and dual users) from 1996 to 2017. We performed a separate validation for total SLT (exclusive SLT and dual users) from 2011-2016. We also considered confidence intervals from each of the surveys.

###  Assessing the Effect of Tobacco Control Policies

 Upon validating the model, we estimate the effect of three policy scenarios (status-quo, counterfactual, and future) for their impacts on exclusive cigarette, exclusive SLT, and dual user prevalence and tobacco-attributable deaths. The *status quo *scenario incorporates policies implemented from 1993 to 2018. The counterfactual scenario represents trends in the absence of any policy change and is programmed with all policies at their 1993 levels. We estimate the impact of policies through 2018 by calculating the relative difference between the counterfactual and status-quo scenarios. The contribution of an individual policy is based on programming *SimSmoke* changes through 2018 on the selected policy with other policies at the 1993 level. The effect of individual policies is measured relative to the summed effects of all individual policies.

 We estimate the potential impact of future policies on tobacco prevalence by increasing all policies to their highest levels as shown in Table 1. The effects are presented relative to the status quo level in the same year for smoking and SLT prevalence and deaths averted.

 For the effect of past and future policies, we also estimate lower and upper credible ranges of the policy effect sizes. Credible ranges are based on the potential minimum and maximum effect of the policy based on reported effects from the literature review.^62^ For cigarettes, they are modeling as +/-50% of the original policy estimated effect, except +/- 25% for taxes. For SLT estimates, we use +/-75% and +/-50% for taxes.

## Results

###  Model Validation 

 Validation comparing *SimSmoke* estimates to the TUS-CPS are in Table 2. For the total adult population, *SimSmoke* predicts a decrease in exclusive male (female) cigarette prevalence from 34.2% (29.5%) in 1993 to 21.2% (21.2%) in 2015 compared to a decrease from 34.6% (29.5%) to 20.8% (19.5%) in 2015 from the TUS-CPS. Estimated *SimSmoke*prevalence from 2002/2003 2006/2007 and 2010/2011 fell within the 95% confidence intervals (CIs) in TUS-CPS data. For male dual users, *SimSmoke* predicts a decrease in prevalence from 2.1% in 1993 to 1.6% in 2015 (22% relative reduction), while TUS-CPS show a decrease from 2.0% in 1993 to 1.1% in 2015 (45% relative reduction), but dual use increases between 2007 (0.9%) and 2010 (1.3%). Dual user predictions for all years fell within the 95% confidence intervals of the reported data.

**Table 2:** Validation: Exclusive Cigarette, SLT, Dual Use, SimSmoke Projections vs. TUS-CPS, by Age and Gender, 1993-2015

**Male Exclusive Smokers**									
**Ages**		**1993**	**2002**	1993-2002*	**2007**	**2010**	**2015**	2002-2015*	1993-2015*
**18+**	**SimSmoke**	34.2%	27.9%	-18.6%	25.3%	22.7%	21.2%	-24.1%	-38%
	**TUS-CPS**	34.6%	27.6%	-20.3%	28.4%	23.2%	20.8%	-24.8%	-40%
	**95%CI**	(31.8%, 37.5%)	(24.9%, 30.4%)		(23.9%, 32.9%)	(20.8%, 25.7%)	(18.5%, 23.3%)		
**18-24**	**SimSmoke**	31.5%	23.7%	-24.7%	21.6%	20.0%	19.9%	-16.3%	-37%
	**TUS-CPS**	30.9%	43.8%	41.9%	27.5%	21.7%	21.0%	-52.1%	-32%
	**95%CI**	(21.1%, 40.7%)	(32.8%, 54.9%)		(11.4%, 43.5%)	(14.6%, 30.8%)	(12.7%, 32.6%)		
**25-44**	**SimSmoke**	38.0%	32.0%	-15.9%	28.8%	25.7%	24.2%	-24.2%	-36%
	**TUS-CPS**	38.3%	32.3%	-15.6%	32.9%	26.3%	21.6%	-33.2%	-44%
	**95%CI**	(34.0%, 42.6%)	(27.6%, 37.0%)		(25.1%, 40.7%)	(22.3%, 30.8%)	(17.6%, 26.2%)		
**45-64**	**SimSmoke**	36.7%	29.6%	-19.3%	26.6%	23.9%	21.7%	-26.5%	-41%
	**TUS-CPS**	37.1%	27.8%	-25.1%	28.9%	26.1%	25.8%	-7.2%	-30%
	**95%CI**	(31.8%, 42.5%)	(23.2%, 32.4%)		(21.6%, 36.3%)	(22.2%, 30.4%)	(21.9%, 30.1%)		
**65+**	**SimSmoke**	21.3%	17.0%	-19.9%	16.5%	15.4%	14.8%	-13.0%	-30%
	**TUS-CPS**	22.2%	11.1%	-50.2%	18.6%	11.7%	11.3%	2.4%	-49%
	**95%CI**	(16.1%, 28.3%)	(6.6%, 15.5%)		(9.5%, 27.7%)	(8.0%, 16.7%)	(8.1%, 15.7%)		
**Female Exclusive Smokers**									
**Ages**		**1993**	**2002**	1993-2002*	**2007**	**2010**	**2015**	2002-2015*	1993-2015*
**18+**	**SimSmoke**	29.5%	25.9%	-12.3%	24.2%	22.2%	21.2%	-18.2%	-28%
	**TUS-CPS**	29.5%	24.7%	-16.5%	26.7%	23.5%	19.5%	-20.9%	-34%
	**95%CI**	(27.2%, 31.8%)	(22.4%, 27.0%)		(23.1%, 30.4%)	(21.4%, 25.7%)	(17.5%, 21.8%)		
**18-24**	**SimSmoke**	34.9%	27.1%	-22.2%	25.3%	23.4%	23.4%	-13.8%	-33%
	**TUS-CPS**	38.5%	37.8%	-1.7%	29.7%	30.4%	16.7%	-55.9%	-57%
	**95%CI**	(30.6%, 46.3%)	(29.1%, 46.5%)		(17.9%, 41.6%)	(22.3%, 39.9%)	(10.5%, 25.4%)		
**25-44**	**SimSmoke**	36.1%	32.8%	-9.3%	30.5%	27.6%	25.9%	-20.9%	-28%
	**TUS-CPS**	35.6%	30.8%	-13.5%	32.8%	26.9%	24.4%	-20.7%	-31%
	**95%CI**	(31.9%, 39.3%)	(26.9%, 34.7%)		(26.1%, 39.6%)	(23.2%, 30.8%)	(20.5%, 28.9%)		
**45-64**	**SimSmoke**	29.2%	26.1%	-10.6%	24.3%	22.3%	21.1%	-19.1%	-28%
	**TUS-CPS**	29.5%	22.5%	-23.6%	28.5%	26.9%	23.6%	4.7%	-20%
	**95%CI**	(25.1%, 34.0%)	(18.8%, 26.3%)		(22.4%, 34.6%)	(23.4%, 30.7%)	(20.0%, 27.6%)		
**65+**	**SimSmoke**	12.4%	11.5%	-6.9%	12.5%	12.7%	13.0%	13.0%	5%
	**TUS-CPS**	12.4%	9.5%	-23.4%	11.5%	10.8%	9.0%	-5.8%	-28%
	**95%CI**	(8.7%, 16.1%)	(5.9%, 13.1%)		(5.5%, 17.4%)	(8.0%, 14.6%)	(6.4%, 12.4%)		
**Male Dual Use**									
**Ages**		**1993**	**2002**	1993-2002*	**2007**	**2010**	**2015**	2002-2015*	1993-2015*
**18+**	**SimSmoke**	2.1%	1.8%	-13.0%	1.7%	1.6%	1.6%	-10.7%	-22%
	**TUS-CPS**	2.0%	1.2%	-39.6%	0.9%	1.3%	1.1%	-9.7%	-45%
	**95%CI**	(1.1%, 2.8%)	(0.5%, 1.9%)		(0.0%, 1.9%)	(0.8%, 2.1%)	(0.6%, 1.9%)		
**18-24**	**SimSmoke**	3.4%	2.1%	-38.1%	2.1%	2.0%	2.0%	-3.6%	-40%
	**TUS-CPS**	3.8%	2.0%	-46.9%	0.0%	2.1%	4.8%	139.9%	27%
	**95%CI**	(0.1%, 7.5%)	(0.0%, 4.9%)		(0.0%, 0.0%)	(0.6%, 7.2%)	(1.7%, 13.3%)		
**25-44**	**SimSmoke**	2.5%	2.7%	10.1%	2.5%	2.2%	2.0%	-25.6%	-18%
	**TUS-CPS**	2.5%	1.9%	-23.9%	1.4%	2.9%	2.0%	3.7%	-21%
	**95%CI**	(1.1%, 3.9%)	(0.5%, 3.3%)		(-0.6%, 3.3%)	(1.7%, 5.1%)	(1.0%, 4.1%)		
**45-64**	**SimSmoke**	0.7%	1.0%	40.3%	1.3%	1.5%	1.6%	69.6%	138%
	**TUS-CPS**	0.5%	0.6%	17.0%	1.1%	0.2%	0.5%	-26.1%	-14%
	**95%CI**	(-0.3%, 1.3%)	(-0.2%, 1.4%)		(-0.6%, 2.7%)	(0.0%, 1.3%)	(0.1%, 1.7%)		
**65+**	**SimSmoke**	2.0%	0.8%	-61.2%	0.5%	0.3%	0.4%	-52.9%	-82%
	**TUS-CPS**	2.1%	0.4%	-80.2%	0.0%	0.0%	0.0%	-100.0%	-100%
	**95%CI**	(0.0%, 4.1%)	(0.0%, 1.2%)		(0.0%, 0.0%)	(0.0%, 1.8%)	(0.0%, 1.4%)		
**Male Exclusive Smokeless Tobacco Use**									
**Ages**		**1993**	**2002**	1993-2002*	**2007**	**2010**	**2015**	2002-2015*	1993-2015*
**18+**	**SimSmoke**	7.1%	6.1%	-12.9%	5.9%	5.6%	5.4%	-12.2%	-23%
	**TUS-CPS**	6.8%	4.1%	-39.0%	3.5%	4.0%	4.7%	14.3%	-30%
	**95%CI**	(5.3%, 8.3%)	(2.9%, 5.4%)		(1.7%, 5.3%)	(3.0%, 5.2%)	(3.7%, 6.2%)		
**18-24**	**SimSmoke**	10.4%	7.2%	-30.9%	7.6%	7.8%	7.9%	10.3%	-24%
	**TUS-CPS**	12.0%	4.9%	-59.4%	0.0%	4.1%	4.8%	-0.6%	-60%
	**95%CI**	(5.1%, 18.9%)	(0.1%, 9.7%)		(0.0%, 0.0%)	(1.6%, 10.1%)	(1.7%, 13.3%)		
**25-44**	**SimSmoke**	6.9%	7.0%	1.5%	6.6%	6.2%	5.8%	-17.1%	-16%
	**TUS-CPS**	6.7%	4.8%	-28.9%	4.9%	4.6%	6.0%	24.7%	-11%
	**95%CI**	(4.5%, 9.0%)	(2.7%, 6.9%)		(1.3%, 8.5%)	(3.0%, 7.1%)	(3.9%, 9.0%)		
**45-64**	**SimSmoke**	5.1%	5.1%	-1.1%	5.0%	4.9%	4.9%	-3.8%	-5%
	**TUS-CPS**	4.9%	2.9%	-41.2%	2.0%	3.0%	4.3%	52.2%	-11%
	**95%CI**	(2.5%, 7.2%)	(1.1%, 4.6%)		(-0.3%, 4.3%)	(1.7%, 5.0%)	(2.8%, 6.7%)		
**65+**	**SimSmoke**	7.6%	5.2%	-32.0%	4.4%	4.0%	3.8%	-26.2%	-50%
	**TUS-CPS**	7.9%	5.0%	-36.9%	5.2%	4.7%	3.8%	-24.5%	-52%
	**95%CI**	(3.9%, 11.9%)	(1.9%, 8.1%)		(0.0%, 10.5%)	(2.6%, 8.4%)	(2.1%, 6.8%)		

Abbreviations: SLT, smokeless tobacco; TUS-CPS, Tobacco Use Supplement of the Current Population Survey.

*SimSmoke* predicts male exclusive SLT prevalence to continuously decline by 23% from 7.1% to 5.4% from 1993 to 2015, while TUS-CPS reports a reduction from 6.8% to 3.5% from 1993 to 2007, but a 35% increase from 3.5% to 4.7% between 2007 and 2015.Validation by age and gender also perform relatively well (see Supplementary Report^17^).

 In a validation against the BRFSS (see Table 3c of Supplementary Report^17^), male (female) smokers from the BRFSS shows a 20.6% (24.3%) relative reduction between 1996 and 2017 compared to a 38.2% (31.0%) relative reduction predicted by *SimSmoke*. SimSmoke predictions fell within the BRFSS 95% CIs before 2011and lower than the intervals since 2011 for both genders. SLT use from the BRFSS shows a 7.1% (33.3%) relative increase between 2011 and 2016 compared to a 3.4% (13.0%) relative reduction predicted by *SimSmoke*, with all the SimSmoke predictions below the BRFSS 95% CI lower bounds.

###  The Effect of Policies Implemented Through 2018

 Results comparing the *status-quo* scenario (ie, policies implemented between 1993 and 2018) to the counterfactual scenario (ie, policies set at 1993 level) are in Table 3 for smoking and SLT prevalence and Table 4 for averted tobacco-attributable deaths.

 Compared to the counterfactual, *SimSmoke* projects that exclusive cigarette prevalence was reduced in relative terms by 23.7% (with credible range of 16.7%, 30.4%) for males and 23.0% (16.2%, 29.4%) for females by 2018. The 2018 reduction for male dual users was 16.4% (11.6%, 21.2%) and for exclusive male SLT users was 4.9% (1.2%, 8.6%). By 2060, the model projects a relative reduction of 32.7% (23.0%, 41.6%) for male and 32.6% (23.1%, 41.4%) for female exclusive smokers, 20.2% (14.0%, 26.6%) for male dual users, and 0.9% (-1.8%, 3.2%) for male and 12.1% (10.0%, 14.0%) for female exclusive SLT users. Compared to the counterfactual, *SimSmoke* estimates that 9018 (6336, 11 601) total tobacco-attributable deaths are averted (64% male deaths averted) by 2018, increasing to 89 547 (62 704, 114 612) averted deaths by 2060.

**Table 3:** Prevalence of Cigarette/SLT/Dual Use in Each Policy Scenario* and the Relative Difference Compared with Counterfatural Scenario,** 1993-2060

**Scenario**		**1993**	**2018**	**2040**	**2060**	**Relative Difference Compared to Counterfactual in 2040**	**Relative Difference Compared to Counterfactual in 2060**
**Male**							
Counter-factual	CIG	34.2%	26.4%	22.9%	22.2%	-	-
	Range	-	(26.4%, 26.4%)	(22.9%, 22.9%)	(22.2%, 22.2%)	-	-
	Dual	2.1%	1.9%	1.7%	1.6%	-	-
	Range	-	(1.9%, 1.9%)	(1.7%, 1.7%)	(1.6%, 1.6%)	-	-
	SLT	7.1%	5.6%	4.8%	4.5%	-	-
	Range	-	(5.6%, 5.6%)	(4.8%, 4.8%)	(4.5%, 4.5%)	-	-
Status quo	CIG	34.2%	20.2%	15.8%	14.9%	-30.7%	-32.7%
	Range	-	(22.0%, 18.4%)	(17.9%, 13.9%)	(17.1%, 12.9%)	(-21.6%, -39.2%)	(-23.0%, -41.6%)
	Dual	2.1%	1.6%	1.4%	1.3%	-19.7%	-20.2%
	Range	-	(1.7%, 1.5%)	(1.5%, 1.3%)	(1.4%, 1.2%)	(-13.6%, -25.7%)	(-14.0%, -26.6%)
	SLT	7.1%	5.3%	4.7%	4.5%	-1.5%	0.9%
	Range	-	(5.5%, 5.1%)	(4.9%, 4.6%)	(4.6%, 4.4%)	(1.1%, -4.3%)	(2.8%, -1.4%)
Price alone	CIG	34.2%	21.8%	17.7%	16.9%	-22.4%	-23.7%
	Range	-	(22.9%, 20.8%)	(18.9%, 16.6%)	(18.1%, 15.8%)	(-17.3%, -27.2%)	(-18.4%, -28.8%)
	Dual	2.1%	1.6%	1.5%	1.4%	-14.9%	-15.1%
	Range	-	(1.7%, 1.6%)	(1.5%, 1.4%)	(1.4%, 1.3%)	(-11.3%, -18.4%)	(-11.5%, -18.8%)
	SLT	7.1%	5.4%	4.9%	4.7%	2.6%	5.4%
	Range	-	(5.5%, 5.4%)	(4.9%, 5.0%)	(4.7%, 4.8%)	(2.1%, 3.1%)	(3.7%, 7.1%)
Smoke-free air law alone	CIG	34.2%	25.8%	22.2%	21.5%	-2.8%	-2.9%
	Range	-	(26.1%, 25.4%)	(22.5%, 21.9%)	(21.9%, 21.2%)	(-1.4%, -4.2%)	(-1.4%, -4.3%)
	Dual	2.1%	1.9%	1.7%	1.6%	-0.4%	-0.3%
	Range	-	(1.9%, 1.9%)	(1.7%, 1.7%)	(1.6%, 1.6%)	(-0.2%, -0.6%)	(-0.1%, -0.4%)
	SLT	7.1%	5.6%	4.8%	4.5%	0.2%	0.3%
	Range	-	(5.6%, 5.6%)	(4.8%, 4.8%)	(4.5%, 4.5%)	(0.1%, 0.2%)	(0.1%, 0.4%)
Media campaign alone	CIG	34.2%	26.3%	22.8%	22.2%	-0.1%	0.0%
	Range	-	(26.4%, 26.3%)	(22.8%, 22.8%)	(22.2%, 22.2%)	(-0.1%, -0.2%)	(0.0%, 0.0%)
	Dual	2.1%	1.9%	1.7%	1.6%	-0.1%	0.0%
	Range	-	(1.9%, 1.9%)	(1.7%, 1.7%)	(1.6%, 1.6%)	(-0.1%, -0.2%)	(0.0%, 0.0%)
	SLT	7.1%	5.6%	4.8%	4.5%	-0.1%	0.0%
	Range	-	(5.6%, 5.5%)	(4.8%, 4.8%)	(4.5%, 4.5%)	(0.0%, -0.2%)	(0.0%, 0.0%)
Cessation treatment alone	CIG	34.2%	25.8%	22.3%	21.7%	-2.4%	-2.1%
	Range	-	(26.1%, 25.5%)	(22.6%, 22.1%)	(21.9%, 21.5%)	(-1.2%, -3.5%)	(-1.1%, -3.2%)
	Dual	2.1%	1.8%	1.7%	1.6%	-1.9%	-1.6%
	Range	-	(1.9%, 1.8%)	(1.7%, 1.7%)	(1.6%, 1.6%)	(-0.9%, -2.7%)	(-0.8%, -2.4%)
	SLT	7.1%	5.5%	4.7%	4.4%	-1.5%	-1.3%
	Range	-	(5.5%, 5.4%)	(4.8%, 4.7%)	(4.5%, 4.4%)	(-0.4%, -2.5%)	(-0.4%, -2.2%)
Health warning alone	CIG	34.2%	26.4%	22.9%	22.2%	0.0%	0.0%
	Range	-	(26.4%, 26.4%)	(22.9%, 22.9%)	(22.2%, 22.2%)	(0.0%, 0.0%)	(0.0%, 0.0%)
	Dual	2.1%	1.9%	1.7%	1.6%	0.0%	0.0%
	Range	-	(1.9%, 1.9%)	(1.7%, 1.7%)	(1.6%, 1.6%)	(0.0%, 0.0%)	(0.0%, 0.1%)
	SLT	7.1%	5.5%	4.7%	4.4%	-1.5%	-1.6%
	Range	-	(5.5%, 5.5%)	(4.8%, 4.7%)	(4.5%, 4.4%)	(-0.8%, -2.3%)	(-0.8%, -2.3%)
Youth access alone	CIG	34.2%	25.7%	21.6%	20.7%	-5.6%	-6.8%
	Range	-	(26.1%, 25.2%)	(22.3%, 20.8%)	(21.5%, 19.7%)	(-2.6%, -9.1%)	(-3.2%, -11.1%)
	Dual	2.1%	1.9%	1.7%	1.6%	-2.8%	-3.5%
	Range	-	(1.9%, 1.8%)	(1.7%, 1.6%)	(1.6%, 1.5%)	(-1.3%, -4.6%)	(-1.6%, -5.8%)
	SLT	7.1%	5.5%	4.8%	4.5%	-0.6%	-1.0%
	Range	34.2%	(5.6%, 5.5%)	(4.8%, 4.7%)	(4.5%, 4.4%)	(0.4%, -1.8%)	(0.4%, -2.5%)
**Female**							
Counter-factual	CIG	29.5%	26.5%	23.7%	23.2%	-	-
	Range	-	(26.5%, 26.5%)	(23.7%, 23.7%)	(23.2%, 23.2%)	-	-
	Dual	0.0%	0.0%	0.0%	0.0%	-	-
	Range	-	(0.0%, 0.0%)	(0.0%, 0.0%)	(0.0%, 0.0%)	-	-
	SLT	0.5%	0.1%	0.1%	0.1%	-	-
	Range	-	(0.1%, 0.1%)	(0.1%, 0.1%)	(0.1%, 0.1%)	-	-
Status quo	CIG	29.5%	20.4%	16.6%	15.6%	-30.1%	-32.6%
	Range	-	(22.2%, 18.7%)	(18.7%, 14.6%)	(17.8%, 13.6%)	(-21.3%, -38.4%)	(-23.1%, -41.4%)
	Dual	0.0%	0.0%	0.0%	0.0%	-	-
	Range	-	(0.0%, 0.0%)	(0.0%, 0.0%)	(0.0%, 0.0%)	-	-
	SLT	0.5%	0.1%	0.1%	0.1%	6.2%	12.1%
	Range	-	(0.1%, 0.1%)	(0.1%, 0.1%)	(0.1%, 0.1%)	(5.6%, 6.7%)	(9.5%, 14.5%)
Price alone	CIG	29.5%	21.9%	18.4%	17.5%	-22.5%	-24.4%
	Range	-	(22.9%, 20.8%)	(19.6%, 17.2%)	(18.8%, 16.3%)	(-17.4%, -27.4%)	(-18.9%, -29.6%)
	Dual	0.0%	0.0%	0.0%	0.0%	-	-
	Range	-	(0.0%, 0.0%)	(0.0%, 0.0%)	(0.0%, 0.0%)	-	-
	SLT	0.5%	0.1%	0.1%	0.1%	7.4%	12.5%
	Range	-	(0.1%, 0.1%)	(0.1%, 0.1%)	(0.1%, 0.1%)	(5.8%, 8.9%)	(9.3%, 15.6%)
Smoke-free air law alone	CIG	29.5%	25.8%	23.1%	22.6%	-2.7%	-2.7%
	Range	-	(26.2%, 25.5%)	(23.4%, 22.8%)	(22.9%, 22.3%)	(-1.3%, -4.0%)	(-1.3%, -4.0%)
	Dual	0.0%	0.0%	0.0%	0.0%	-	-
	Range	-	(0.0%, 0.0%)	(0.0%, 0.0%)	(0.0%, 0.0%)	-	-
	SLT	0.5%	0.1%	0.1%	0.1%	0.6%	1.0%
	Range	-	(0.1%, 0.1%)	(0.1%, 0.1%)	(0.1%, 0.1%)	(0.3%, 0.9%)	(0.5%, 1.5%)
Media campaign alone	CIG	29.5%	26.4%	23.7%	23.2%	-0.2%	0.0%
	Range	-	(26.4%, 26.3%)	(23.7%, 23.7%)	(23.2%, 23.2%)	(-0.1%, -0.2%)	(0.0%, 0.0%)
	Dual	0.0%	0.0%	0.0%	0.0%	-	-
	Range	-	(0.0%, 0.0%)	(0.0%, 0.0%)	(0.0%, 0.0%)	-	-
	SLT	0.5%	0.1%	0.1%	0.1%	-0.1%	0.0%
	Range	-	(0.1%, 0.1%)	(0.1%, 0.1%)	(0.1%, 0.1%)	(0.0%, -0.1%)	(0.0%, 0.0%)
Cessation treatment alone	CIG	29.5%	25.9%	23.2%	22.8%	-2.1%	-1.8%
	Range	-	(26.2%, 25.6%)	(23.5%, 23.0%)	(23.0%, 22.6%)	(-1.1%, -3.1%)	(-0.9%, -2.6%)
	Dual	0.0%	0.0%	0.0%	0.0%	-	-
	Range	-	(0.0%, 0.0%)	(0.0%, 0.0%)	(0.0%, 0.0%)	-	-
	SLT	0.5%	0.1%	0.1%	0.1%	-1.6%	-1.4%
	Range	-	(0.1%, 0.1%)	(0.1%, 0.1%)	(0.1%, 0.1%)	(-0.4%, -2.7%)	(-0.4%, -2.5%)
Health warning alone	CIG	29.5%	26.5%	23.7%	23.2%	0.0%	0.0%
	Range	-	(26.5%, 26.5%)	(23.7%, 23.7%)	(23.2%, 23.2%)	(0.0%, 0.0%)	(0.0%, 0.0%)
	Dual	0.0%	0.0%	0.0%	0.0%	-	-
	Range	-	(0.0%, 0.0%)	(0.0%, 0.0%)	(0.0%, 0.0%)	-	-
	SLT	0.5%	0.1%	0.1%	0.1%	-1.7%	-1.7%
	Range	-	(0.1%, 0.1%)	(0.1%, 0.1%)	(0.1%, 0.1%)	(-0.9%, -2.6%)	(-0.9%, -2.6%)
Youth access alone	CIG	29.5%	25.9%	22.6%	21.7%	-5.0%	-6.4%
	Range	-	(26.2%, 25.5%)	(23.2%, 21.8%)	(22.5%, 20.8%)	(-2.3%, -8.0%)	(-3.0%, -10.3%)
	Dual	0.0%	0.0%	0.0%	0.0%	-	-
	Range	-	(0.0%, 0.0%)	(0.0%, 0.0%)	(0.0%, 0.0%)	-	-
	SLT	0.5%	0.1%	0.1%	0.1%	2.0%	3.0%
	Range	-	(0.1%, 0.1%)	(0.1%, 0.1%)	(0.1%, 0.1%)	(0.9%, 3.2%)	(1.4%, 4.9%)

Abbreviations: CIG, cigarette; SLT, smokeless tobacco; Dual, dual use of cigarette and smokeless tobacco. *The reported prevalence is the best estimate with its left/right bound range from model predictions using lower/upper policy effect sizes. **Policies fixed at 1993 level.

**Table 4:** Tobacco-Attributable Deaths and Deaths averted* for Both Genders in Each Individual Policy Scenario, 1993-2060

**Male**							
**Tobacco-attributable deaths**		**1993**	**2018**	**2040**	**2060**	**Cumulative** **Through 2040**	**Cumulative** **Through 2060**
Status quo	CIG	4554	5359	4503	3607	245 372	324 358
	Range	-	(5546, 5181)	(4830, 4199)	(4039, 3210)	(252 977, 238 192)	(339 558, 310 176)
	Dual	352	240	348	296	13 476	19 942
	Range	-	(247, 234)	(366, 331)	(317, 275)	(13 818, 13 147)	(20 682, 19 225)
	SLT	189	167	201	177	8839	12 661
	Range	-	(172, 163)	(209, 193)	(184, 169)	(9033, 8653)	(13 020, 12 314)
	Total	5096	5767	5052	4080	267 688	356 960
	Range	-	(5964, 5578)	(5405, 4723)	(4541, 3655)	(275 828, 259 992)	(373 260, 341 715)
Counter-factual	CIG	4554	5974	5601	5082	270 599	375 500
	Range	-	(5974, 5974)	(5601, 5601)	(5082, 5082)	(270 599, 270 599)	(375 500, 375 500)
	Dual	352	263	408	366	14 637	22 441
	Range	-	(263, 263)	(408, 408)	(366, 366)	(14 637, 14 637)	(22 441, 22 441)
	SLT	189	176	215	187	9199	13 287
	Range	-	(176, 176)	(215, 215)	(187, 187)	(9199, 9199)	(13 287, 13 287)
	Total	5096	6413	6225	5636	294 434	411 228
	Range	-	(6413, 6413)	(6225, 6225)	(5636, 5636)	(294 434, 294 434)	(411 228, 411 228)
**Deaths averted**		**1993**	**2018**	**2040**	**2060**	**Cumulative through 2040**	**Cumulative Through 2060**
Status quo	CIG	0	615	1098	1475	25 227	51 142
	Range	-	(428, 793)	(771, 1402)	(1043, 1872)	(17 622, 32 407)	(35 941, 65 323)
	Dual	0	23	60	70	1160	2499
	Range	-	(16, 29)	(42, 77)	(49, 91)	(818, 1489)	(1759, 3216)
	SLT	0	9	14	11	359	626
	Range	-	(4, 13)	(6, 22)	(3, 18)	(166, 546)	(268, 973)
	Total	0	646	1173	1555	26 746	54 267
	Range	-	(448, 835)	(819, 1501)	(1095, 1981)	(18 606, 34 442)	(37 967, 69 512)
Price alone	CIG	0	455	798	1081	18 545	37 587
	Range	-	(348, 560)	(615, 970)	(836, 1311)	(14 214, 22 697)	(28 933, 45 812)
	Dual	0	18	46	53	910	1938
	Range	-	(14, 22)	(35, 57)	(40, 65)	(694, 1119)	(1478, 2382)
	SLT	0	7	8	2	257	375
	Range	-	(4, 10)	(4, 12)	(1, 4)	(130, 381)	(185, 561)
	Total	0	481	852	1136	19 712	39 900
	Range	-	(365, 592)	(654, 1039)	(877, 1381)	(15 038, 24 197)	(30 596, 48 755)
Smoke free air law alone	CIG	0	72	129	137	2940	5586
	Range	-	(36, 108)	(65, 192)	(69, 204)	(1478, 4386)	(2809, 8330)
	Dual	0	0	0	0	0	-1
	Range	-	(0, 0)	(0, 0)	(0, 0)	(0, 0)	(0, -1)
	SLT	0	1	2	1	45	83
	Range	-	(0, 1)	(1, 3)	(1, 2)	(22, 67)	(41, 124)
	Total	0	73	131	138	2984	5668
	Range	-	(37, 109)	(66, 196)	(70, 206)	(1500, 4453)	(2850, 8454)
Media campaign alone	CIG	0	34	21	3	976	1200
	Range	-	(17, 51)	(10, 31)	(2, 5)	(488, 1463)	(600, 1800)
	Dual	0	2	2	0	54	72
	Range	-	(1, 2)	(1, 2)	(0, 0)	(27, 81)	(36, 108)
	SLT	0	0	1	0	17	25
	Range	-	(0, 1)	(0, 1)	(0, 0)	(4, 29)	(6, 44)
	Total	0	36	23	4	1,047	1,297
	Range	-	(18, 55)	(11, 35)	(2, 5)	(519, 1574)	(643, 1952)
Cessation treatment alone	CIG	0	89	161	135	3840	6805
	Range	-	(45, 132)	(82, 238)	(69, 199)	(1940, 5703)	(3446, 10 083)
	Dual	0	3	10	7	173	349
	Range	-	(1, 4)	(5, 14)	(4, 11)	(87, 258)	(176, 518)
	SLT	0	1	4	4	70	151
	Range	-	(0, 2)	(1, 7)	(1, 7)	(18, 122)	(38, 261)
	Total	0	93	174	146	4084	7305
	Range	-	(47, 139)	(88, 259)	(73, 217)	(2045, 6082)	(3660, 10 862)
Health warning alone	CIG	-	-	-	-	-	-
	Range						
	Dual	-	-	-	-	-	-
	Range						
	SLT	0	1	3	3	48	111
	Range	-	(0, 1)	(1, 4)	(2, 5)	(24, 72)	(56, 166)
	Total	0	1	3	2	46	95
	Range	-	(0, 1)	(1, 4)	(1, 3)	(23, 69)	(48, 141)
Youth access alone	CIG	0	4	82	250	809	4,111
	Range	-	(2, 5)	(39, 130)	(118, 400)	(387, 1276)	(1952, 6538)
	Dual	0	0	3	10	33	163
	Range	-	(0, 0)	(2, 5)	(5, 16)	(16, 53)	(77, 264)
	SLT	0	0	0	1	3	17
	Range	-	(0, 0)	(0, 1)	(0, 3)	(0, 7)	(0, 39)
	Total	0	4	86	261	845	4,292
	Range	-	(2, 6)	(41, 136)	(122, 418)	(403, 1336)	(2026, 6841)
**Female**							
**Tobacco-Attributable Deaths**		**1993**	**2018**	**2040**	**2060**	**Cumulative** **Through 2040**	**Cumulative** **Through 2060**
Status quo	CIG	2313	3347	3343	2531	150 680	208 390
	Range	-	(3464, 3235)	(3576, 3126)	(2842, 2246)	(155 714, 145 918)	(218 892, 198 608)
	SLT	31	10	8	6	777	905
	Range	-	(10, 10)	(8, 8)	(6, 6)	(791, 764)	(921, 889)
	Total	2344	3357	3351	2537	151 457	209 295
	Range	-	(3474, 3245)	(3584, 3133)	(2848, 2252)	(156 505, 146 682)	(219 813, 199 497)
Counter-factual	CIG	2313	3732	4116	3613	167 225	243 642
	Range	-	(3732, 3732)	(4116, 4116)	(3613, 3613)	(167 225, 167 225)	(243 642, 243 642)
	SLT	31	11	8	6	803	932
	Range	-	(11, 11)	(8, 8)	(6, 6)	(803, 803)	(932, 932)
	Total	2344	3742	4125	3619	168 028	244 574
**Deaths Averted**		**1993**	**2018**	**2040**	**2060**	**Cumulative** **Through 2040**	**Cumulative** **Through 2060**
Status quo	CIG	0	385	774	1082	16 546	35 252
	Range	-	(268, 497)	(540, 991)	(771, 1367)	(11 511, 21 307)	(24 750, 45 034)
	SLT	0	1	0	0	26	28
	Range	-	(0, 1)	(0, 1)	(0, 0)	(12, 39)	(11, 43)
	Total	0	385	774	1082	16 571	35 279
	Range	-	(268, 498)	(540, 992)	(770, 1367)	(11 523, 21 346)	(24 761, 45 077)
Price alone	CIG	0	285	557	809	12 064	25 907
	Range	-	(217, 351)	(429, 678)	(627, 979)	(9234, 14 780)	(19 937, 31 575)
	SLT	0	1	0	0	20	19
	Range	-	(0, 1)	(0, 0)	(0, 0)	(10, 30)	(8, 30)
	Total	0	286	557	809	12 084	25 926
	Range	-	(218, 352)	(429, 679)	(627, 979)	(9245, 14 811)	(19 946, 31 604)
Smoke free air law alone	CIG	0	45	95	95	2021	3931
	Range	-	(23, 67)	(48, 142)	(48, 142)	(1016, 3015)	(1977, 5861)
	SLT	0	0	0	0	0	0
	Range	-	(0, 0)	(0, 0)	(0, 0)	(0, 0)	(0, 0)
	Total	0	45	95	95	2021	3930
	Range	-	(23, 67)	(48, 142)	(48, 142)	(1016, 3015)	(1977, 5861)
Media camp-aign alone	CIG	0	23	20	5	706	962
	Range	-	(11, 34)	(10, 30)	(3, 8)	(353, 1059)	(481, 1443)
	SLT	0	0	0	0	1	2
	Range	-	(0, 0)	(0, 0)	(0, 0)	(0, 2)	(0, 3)
	Total	0	23	20	5	708	963
	Range	-	(11, 34)	(10, 30)	(3, 8)	(354, 1062)	(481, 1446)
Cessation treatment alone	CIG	0	56	130	109	2706	5153
	Range	-	(28, 83)	(66, 193)	(55, 161)	(1367, 4020)	(2608, 7635)
	SLT	0	0	0	0	4	6
	Range	-	(0, 0)	(0, 0)	(0, 0)	(1, 7)	(2, 11)
	Total	0	56	130	109	2711	5159
	Range	-	(28, 83)	(66, 193)	(55, 161)	(1368, 4027)	(2610, 7646)
Health warning alone	CIG	-	-	-	-	-	-
	Range						
	SLT	0	0	0	0	3	5
	Range	-	(0, 0)	(0, 0)	(0, 0)	(1, 4)	(2, 7)
	Total	0	0	0	0	3	5
	Range	-	(0, 0)	(0, 0)	(0, 0)	(1, 4)	(2, 7)
Youth access alone	CIG	0	0	0	0	0	0
	Range	-	(0, 0)	(0, 0)	(0, 0)	(0, 0)	(0, 0)
	SLT	0	0	0	0	0	0
	Range	-	(0, 0)	(0, 0)	(0, 0)	(0, 0)	(0, 0)
	Total	0	0	0	0	0	0
	Range	-	(0, 0)	(0, 0)	(0, 0)	(0, 0)	(0, 0)

Abbreviations: CIG, cigarette; SLT, smokeless tobacco; Dual, dual use of cigarette and smokeless tobacco. *The reported deaths averted is given as the best estimate with its left/right bound range from model predictions using lower/upper policy effect sizes.

 By individual policy, increased prices (largely due to cigarette taxes) reduce exclusive cigarette use rates by an average of 17.5% for males and females by 2018 and avert 65,826 total deaths by 2060. Smoke-free air laws yield a 2.5% reduction in exclusive cigarette use by 2018 and avert 9598 deaths by 2060. Cessation treatment policies show a relative reduction of 2.2% in 2018, with 12 464 averted deaths by 2060. Youth access restrictions show a 2.6% reduction in exclusive cigarette use and avert 6530 deaths by 2060.

*SimSmoke* estimated the highest reduction in 2018 exclusive smokers’ prevalence for taxation (68%), followed by youth access policy (11%), smoke-free air laws (10%), cessation treatments (9%), and tobacco control campaigns (2%). For male dual prevalence, impacts were higher for taxation (76%), cessation treatment (10%), and youth access enforcement (9%) but generally lower for other policies. For SLT use, the order is taxes (44%), cessation programs (24%), health warnings (24%), and youth access policy (5%).

###  The Effect of Stronger Future Policies 

Tables 5 and 6 show the projected smoking prevalence and exclusive SLT use from strengthening tobacco control policies. Increasing the excise tax per pack of cigarettes by $2.00 and SLT by $2.00 reduces the average male and female smoking prevalence by 9.6%, reduces male and female SLT use by 10.3% and 9.6% initially increasing to 18.5% and 12.7% by 2060, averting 11,072 deaths by 2060. An SLT tax increase of $2.00 yields a 10.3% (5.2%, 15.2%) and a 9.6% (4.8%, 14.1%) immediate relative reduction for male and female exclusive SLT users, increasing to 18.5% and 12.7% by 2060 and averting 615 deaths. Increasing smoke-free air laws to their highest level, as shown in Table 1, reduces overall smoking prevalence by 6.5% for both genders with 12 086 fewer deaths, but with minimal effects on SLT use. A well-funded campaign reduces smoking prevalence by 3.5% with 7188 deaths averted but has limited SLT effects. Strict marketing restrictions reduce cigarette and SLT use by 7% with 9966 smoking and 279 SLT deaths averted. Strong health warnings reduce smoking by 6.5% with 10 205 fewer smoking deaths and reduce SLT use by 4.7% with 192 fewer SLT-related deaths. Cessation policies reduce smoking prevalence by 3.2% with 9249 averted smoking deaths and reduce SLT use by 2.0% with 136 averted SLT-related deaths. With strong youth policies, *SimSmoke* shows a reduction of 7% for cigarette and 1.5% for SLT use, averting 1035 smoking-related and 5 SLT-related deaths by 2060.

**Table 5:** Kentucky Prevalence* of Cigarette/SLT/Dual Use for Future Policy Change Scenarios, 2019-2060

**Males**				
**Overall smoking prevalence**	**2018**	**2019**	**2040**	**2060**
Status quo policies	22% (24%, 20%)	21% (23%, 20%)	17% (19%, 15%)	16% (18%, 14%)
**% Change in Smoking Prevalence From Status Quo**				
Increase tax by $2	-	-5.1% (-3.9%, -6.3%)	-7.8% (-6.0%, -9.6%)	-9.2% (-7.1%, -11.4%)
Comprehensive smoke-free air laws and enforcement	-	-5.1% (-2.5%, -7.6%)	-6.3% (-3.1%, -9.5%)	-6.6% (-3.3%, -10.0%)
Comprehensive marketing ban and enforcement	-	-4.1% (-2.1%, -6.2%)	-6.4% (-3.2%, -9.7%)	-7.6% (-3.8%, -11.5%)
High intensity tobacco control campaigns	-	-2.8% (-1.4%, -4.2%)	-3.6% (-1.8%, -5.4%)	-3.7% (-1.8%, -5.6%)
Strong health warnings	-	-3.0% (-1.5%, -4.6%)	-6.0% (-3.0%, -9.1%)	-7.0% (-3.5%, -10.5%)
Strong youth access enforcement	-	0.0% (0.0%, 0.0%)	-4.2% (-1.7%, -7.5%)	-7.1% (-2.9%, -12.8%)
Cessation treatment policies	-	-2.0% (-1.0%, -3.0%)	-3.7% (-1.7%, -5.1%)	-3.4% (-1.6%, -4.6%)
All above policies with $2 tax increase	-	-23.4% (-13.5%, -32.3%)	-36.9% (-21.3%, -50.4%)	-41.3% (-24.1%,- 55.8%)
**Exclusive SLT Use Prevalence**	**2018**	**2019**	**2040**	**2060**
Status quo policies	5.3% (5.5%, 5.1%)	5.3% (5.5%, 5.1%)	4.7% (4.9%, 4.6%)	4.5% (4.6%, 4.4%)
**% Change in SLT Use Prevalence from Status Quo**				
Increase tax by $2	-	-10.3% (-5.2%, -15.2%)	-15.6% (-8.0%, -22.6%)	-18.5% (-9.6%, -26.6%)
Comprehensive. Smoke free laws and enforcement	-	-0.4% (-0.1%, -0.6%)	-0.3% (0.0%, -0.6%)	-0.2% (0.1%, -0.5%)
Comprehensive marketing ban and enforcement	-	-4.4% (-1.1%, -7.8%)	-6.3% (-1.5%, -11.1%)	-7.3% (-1.7%, -13.0%)
High Intensity tobacco control campaigns	-	-1.2% (-0.3%, -2.2%)	-1.5% (-0.4%, -2.7%)	-1.5% (-0.3%, -2.7%)
Strong health warnings	-	-2.0% (-0.5%, -3.5%)	-4.0% (-0.9%, -7.2%)	-4.8% (-1.1%, -8.6%)
Strong youth access enforcement	-	0.0% (0.0%, 0.0%)	-0.8% (0.1%, -2.1%)	-1.5% (0.2%, -3.8%)
Cessation treatment policies	-	-1.0% (-0.2%, -1.8%)	-2.0% (-0.5%, -3.1%)	-1.9%(-0.5%, -3.0%)
All of the above policies with $2 tax increase	-	-18.6% (-7.4%, -28.6%)	-28.7% (-11.1%, -43.6%)	-32.9% (-12.8%, -49.3%)
**Females**				
**Overall smoking prevalence**	**2018**	**2019**	**2040**	**2060**
Status Quo Policies	20% (22%, 19%)	20% (22%, 18%)	17% (19%, 15%)	16% (18%, 14%)
**% Change in Smoking Prevalence from Status Quo**				
Increase tax by $2	-	-5.1% (-3.9%, -6.3%)	-8.1% (-6.1%, -10.0%)	-10.0% (-7.5%, -12.5%)
Comprehensive. Smoke free laws and enforcement	-	-5.3% (-2.7%, -8.0%)	-6.3% (-3.1%, -9.5%)	-6.5% (-3.2%, -9.8%)
Comprehensive marketing ban and enforcement	-	-4.1% (-2.0%, -6.1%)	-6.0% (-3.0%, -9.0%)	-7.2% (-3.6%, -10.9%)
High intensity tobacco control campaigns	-	-2.8% (-1.4%, -4.2%)	-3.5% (-1.7%, -5.2%)	-3.5% (-1.7%, -5.2%)
Strong health warnings	-	-3.0% (-1.5%, -4.5%)	-5.4% (-2.7%, -8.3%)	-6.3% (-3.1%, -9.5%)
Strong youth access enforcement	-	0.0% (0.0%, 0.0%)	-3.6% (-1.5%, -6.5%)	-6.7% (-2.8%, -12.2%)
Cessation treatment policies	-	-2.0% (-1.0%, -3.0%)	-3.4% (-1.6%, -4.8%)	-2.9% (-1.4%, -3.9%)
All of the above policies with $2 tax increase	-	-23.7% (-15.5%, -31.3%)	-35.6% (-23.0%, -47.0%)	-40.1% (-26.4%, -52.4%)
**Exclusive SLT Use Prevalence**	**2018**	**2019**	**2040**	**2060**
Status quo policies	0.1% (0.1%, 0.1%)	0.1% (0.1%, 0.1%)	0.1% (0.1%, 0.1%)	0.1% (0.1%, 0.1%)
**% Change in SLT Use Prevalence From Status Quo**				
Increase tax by $2	-	-9.6% (-4.8%, -14.1%)	-12.0% (-6.1%, -17.5%)	-12.7% (-6.5%, -18.6%)
Comprehensive. Smoke free laws and enforcement	-	-0.4% (-0.1%, -0.6%)	0.1% (0.2%, -0.1%)	0.8% (0.6%, 0.8%)
Comprehensive marketing ban and enforcement	-	-4.4% (-1.1%, -7.8%)	-6.7% (-1.5%, -12.0%)	-7.1% (-1.2%, -13.1%)
High intensity tobacco control campaigns	-	-1.2% (-0.3%, -2.2%)	-1.4% (-0.3%, -2.6%)	-1.2% (-0.1%, -2.3%)
Strong health warnings	-	-2.0% (-0.5%, -3.5%)	-4.5% (-1.0%, -8.0%)	-4.8% (-0.8%, -8.9%)
Strong youth access enforcement	-	0.0% (0.0%, 0.0%)	0.4% (0.2%, 0.7%)	1.7% (0.8%, 2.7%)
Cessation treatment policies	-	-1.0% (-0.2%, -1.8%)	-2.1% (-0.5%, -3.3%)	-2.1% (-0.5%, -3.3%)
All of the above policies with $2 tax increase	-	-17.9% (-7.1%, -27.7%)	-25.0% (-8.9%, -38.9%)	-24.6% (-7.7%, -39.4%)

Abbreviations: SLT, smokeless tobacco. * The reported prevalence is the best estimate with its left/right bound range from model predictions using lower/upper policy effect sizes.

**Table 6:** Sensitivity Analysis for Lives Saved* (Both Genders) for Stronger Future Policy Scenarios in Kentucky, 2019-2060

**Deaths Averted: All Smokers**				
**Scenario**	**2040**	**2060**	Cumulative** Through 2040	Cumulative** Through 2060
Increase tax by $2	284 (237, 318)	404 (350, 435)	3815 (3143, 4336)	10 457 (8813, 11 604)
Comprehensive. Smoke free laws and enforcement	370 (203, 504)	364 (208, 476)	4783 (2600, 6589)	12 086 (6695, 16 308)
Comprehensive marketing ban	298 (163, 408)	326 (185, 429)	3809 (2063, 5266)	9966 (5503, 13 506)
High intensity tobacco control campaigns	221 (121, 303)	218 (124, 286)	2778 (1505, 3840)	7188 (3966, 9741)
Strong health warnings	307 (167, 424)	344 (195, 452)	3611 (1941, 5027)	10 205 (5606, 13 885)
Strong youth access enforcements	8 (4, 12)	110 (53, 173)	18 (9, 27)	1035 (493, 1618)
Cessation treatment policies	288 (149, 368)	291 (157, 347)	3200 (1650, 4190)	9249 (4843, 11 641)
All above policies with $2 increase in tax	1853 (1139, 2314)	2002 (1326, 2336)	23 206 (14 158, 29 447)	61 626 (38 711, 75 638)
**Deaths Averted: Exclusive SLT Users**				
**Scenario**	**2040**	**2060**	Cumulative** Through 2040	Cumulative** Through 2060
Increase tax by $2	18 (10, 25)	22 (12, 30)	210 (112, 292)	615 (330, 849)
Comprehensive. Smoke free laws and enforcement	1 (0, 1)	1 (0, 1)	8 (2, 14)	23 (6, 39)
Comprehensive marketing ban	8 (2, 13)	10 (3, 16)	97 (26, 160)	279 (74, 458)
High intensity tobacco control campaigns	3 (1, 4)	3 (1, 5)	30 (8, 49)	87 (23, 144)
Strong health warnings	5 (1, 9)	7 (2, 12)	58 (15, 95)	192 (51, 314)
Strong youth access enforcements	0 (0, 0)	0 (0, 1)	0 (0, 0)	5 (0, 11)
Cessation treatment policies	4 (1, 6)	5 (1, 8)	38 (10, 60)	136 (34, 208)
All above policies with $2 increase in tax	37 (15, 54)	45 (19, 64)	426 (173, 619)	1276 (519, 1825)

Abbreviation: SLT, smokeless tobacco. * Lives saved is reported as the best estimate with its left/right bound range from model predictions using lower/upper policy effect sizes. ** Cumulative is reported from 2019 through a particular year.

 With the above policies in combination, *SimSmoke* predicts that by 2060, male and female prevalence are reduced by 41%, male and female exclusive SLT use are reduced by 33% and 25% respectively. Premature deaths are reduced by 61 626 for smoking-attributable deaths and 1276 from exclusive SLT-attributable deaths.

## Discussion

 Our projection of trends in cigarette and dual use from Kentucky *SimSmoke* are relatively well-supported by two large scale nationally representative surveys, TUS-CPS and BRFSS. However, the analysis of SLT does not fit the data as well. *SimSmoke* underestimated the reduction in SLT prevalence in sub-periods 1993-2002 and 2002-2007 in the TUS-CPS and then failed to detect the increase between 2007 and 2015 in the TUS-CPS, and the increase between 2011 and 2017 found from BRFSS.

 Similar to previous literature,^63,64^ our analyses show that Kentucky cigarette and SLT rates decreased between 1993 and 2007, particularly for males. The greater observed than estimated *SimSmoke*reductions in SLT use through 2007 (48.5% in TUS-CPS vs. 17.1% from *SimSmoke*) may reflect the indirect impact of cigarette-targeted tobacco control policies. The estimated effect sizes of cigarette-oriented policies on SLT use in *SimSmoke* reflects studies of use patterns prior to 2007. With major changes in tobacco control policies between 1993 and 2007, these findings suggest that cigarette-oriented policies may have additional impacts on SLT use. Since reductions in cigarette use were associated with reductions in SLT use, these patterns suggest a complementary relationship between the use of these two products. However, since dual use did not increase, these patterns may reflect changes in norms towards single tobacco use rather the dual use of the products.

 Although not predicted by *SimSmoke*, SLT use increased in recent years according to the TUS-CPS and BRFSS estimates and other recent studies.^65-70^ These increases may reflect the growth in SLT marketing by cigarette manufacturers.^71^ The increase in unregulated sales of flavored SLT products also could contribute to smoking initiation at younger ages.^72,73^ Since 2006, cigarette companies Reynolds bought one of the largest SLT companies Conwood, and Altria bought US Smokeless Tobacco Company and both cigarette companies produced their own varieties of SLT (eg, Camel snus).^74^ During that period, cigarette companies increased the marketing of SLT, lowered prices and, in particular, marketed SLT as a product that could be used in places (eg, worksites) where smoking was not allowed.^75,76^ Similar and even stronger patterns of increased SLT use were observed in Minnesota,^77^ which implemented a statewide smoke-free air ban. The increase in unregulated sales of flavored SLT products also could have contributed to SLT initiation at younger ages,^72,73^as indicated by the particularly large increase in exclusive and dual SLT use of young adults (especially ages 18-24). These patterns suggest that SLT use has acted as a substitute for cigarettes in recent years.

 The findings in this study have implications for the recent growth in e-cigarette and heated tobacco product use. From 1993-2002, SLT use fell during a period of active cigarette-oriented policies. This finding suggests that more restrictive cigarette-oriented policies may also serve in a complementary fashion to reduce e-cigarette use. At the same time, e-cigarettes may be a better substitute for cigarettes than SLTs, since they appear to more efficiently deliver nicotine and provide sensorimotor effects closer to cigarettes than other non-combustible tobacco products.^15^ In that case, stronger policies may cause increased substitution of e-cigarettes for cigarettes, especially if e-cigarette policies are weak. In recent years (since 2006), cigarette and SLT companies have been more active in promoting new forms of oral tobacco products.^10,11,78^ The recent growth in SLT use by young adults also suggests that cigarette companies may actively promote e-cigarettes to be used with cigarettes (eg, dual use), again suggesting a complementary relationship. In particular, Altria recently purchased oral tobacco firm “On”^10^ and introduced heated tobacco products (ie, IQOS). With this increasing diversification of products, the landscape for nicotine delivery products is likely to become increasingly complex.^79,80^ It will be important to monitor the behavior of cigarette companies and how they promote products that may serve as an alternative to cigarettes.

 While the current role of SLT use vis-à-vis e-cigarettes needs further policy consideration, there is a potential for further reductions in tobacco usage in Kentucky. For example, a *SimSmoke*model for Minnesota,^62^ one of the leading tobacco control policy states, estimates that policies implemented since 1993 led to a reduction in smoking prevalence of 35% and SLT use by 23% by 2018. By that same year, *SimSmoke* results for Kentucky estimate a smoking prevalence reduction of 24% and 5% for male SLT users. The variations are mostly due to the different levels of policies implemented. By 2018, Minnesota had a cigarette excise tax of $3.04, smoke free-air laws in workplaces, schools, and restaurants and bars, an adequate level of cessation coverage, and spent 32% of the CDC’s recommended budget for tobacco control. In contrast, Kentucky had an excise tax of $0.60 and a third of the state with smoke-free air laws in schools, government buildings, workplaces, and restaurants, and the state spent 10% of the CDC recommended level for tobacco control expenditures.^5^

 Since early 2018, Kentucky has started to move towards more aggressive regulations by implementing a CDC-recommended comprehensive cessation program and increasing the cigarette tax in July 2018. An increase in the Kentucky cigarette excise tax of $0.50 to $1.10^81^ could achieve a reduction in smoking prevalence of 2.8% in the first few years. If the cigarette tax is increased by an additional $0.90 to $2.00, Kentucky would then be above the mean US state taxation of $1.82, which would result in further reducing smoking prevalence by 5.1% by 2060.

 Like all models, our results are only as strong as the assumptions and underlying data. We assumed that projections of cigarette use are based on initiation and cessation rates from 1993, but subject to policy changes over time. Thus, the initiation and cessation rate estimated using smoking prevalence data from 1993, and the policy levels and effect sizes play an important role. Also, we do not explicitly incorporate the role of industry, which may have had a major impact when the major SLT producing firms were acquired by cigarette manufacturers.^74^ In addition, cessation and relapse data were not available for SLT use. Also, we treated SLT as a homogeneous category in terms of risks and an ability to substitute for cigarettes, although new forms, such as snus and other oral tobacco products, have come onto the market.^10,11^

 Another limitation of the model is that it includes only cigarettes and SLT use and does not incorporate the use of other nicotine delivery products, including cigars, water pipes, heated tobacco products, and e-cigarettes. Each may act as substitute or complement to the use of cigarettes and SLT. While use of these products may be a relatively minor contributor to overall tobacco-related harms, policies should be directed at all of these products, particularly small cigars.^82^

 Finally, we assume that cigarette-oriented and SLT-oriented policies have independent effects. As suggested above, cigarette-oriented policies may have indirect impacts on SLT use through social norms. The literature on the inter-relationship between the effects of different types of policies is sparse.^42,62^ While some smokeless demand studies incorporate cigarette prices and some cigarette demand studies incorporate smokeless prices,^40-42,64^ the studies obtain mixed results regarding whether the two products are substitutes or complements.^42^ In general, greater information is needed on the inter-related effects of policies targeting different tobacco products.

## Conclusion

 While the landscape for nicotine delivery products has dramatically changed in the last 10 years, some lessons can be gleaned from our results. First, with cigarettes still the dominant form of nicotine delivery, cigarette-oriented policies appear to be an effective means of reducing the use of nicotine delivery products. However, with SLT use increasing in recent years, policies directed at SLT use may also play a role. With cigarette manufacturers having acquired major SLT firms, it is important to monitor the role of the cigarette industry. With strong incentives to protect the high profits from cigarettes, cigarette firms can be expected to encourage dual use rather than switching to other tobacco products. Well-targeted policies and regulations, such as cigarette and SLT tax increases and media campaigns, will be needed to achieve reductions in SLT use.

## Ethical issues

 None, the study uses publicly available data.

## Competing interests

 Authors declare that they have no competing interests.

## Authors’ contributions

 LMSR wrote and edited much of the original draft. DTL conceived the idea and edited and revised the paper, ZY and YL conducted the modeling, wrote up results and reviewed the manuscript.

## Disclaimer

 The content is solely the responsibility of the authors and does not necessarily represent the official views of the National Institute on Drug Abuse of the National Institutes of Health.

## Funding

 This work was supported by the National Institute on Drug Abuse of the National Institutes of Health under grant R01DA036497.
